# Seismic waveform simulation for models with fluctuating interfaces

**DOI:** 10.1038/s41598-018-20992-z

**Published:** 2018-02-15

**Authors:** Ying Rao, Yanghua Wang

**Affiliations:** 1 State Key Laboratory of Petroleum Resources and Prospecting, China University of Petroleum (Beijing), Beijing, 102249 China; 2 Centre for Reservoir Geophysics, Department of Earth Science and Engineering, Imperial College London, London, SW7 2BP UK

## Abstract

The contrast of elastic properties across a subsurface interface imposes a dominant influence on the seismic wavefield, which includes transmitted and reflected waves from the interface. Therefore, for an accurate waveform simulation, it is necessary to have an accurate representation of the subsurface interfaces within the numerical model. Accordingly, body-fitted gridding is used to partition subsurface models so that the grids coincide well with both the irregular surface and fluctuating interfaces of the Earth. However, non-rectangular meshes inevitably exist across fluctuating interfaces. This non-orthogonality degrades the accuracy of the waveform simulation when using a conventional finite-difference method. Here, we find that a summation-by-parts (SBP) finite-difference method can be used for models with non-rectangular meshes across fluctuating interfaces, and can achieve desirable simulation accuracy. The acute angle of non-rectangular meshes can be relaxed to as low as 47°. The cell size rate of change between neighbouring grids can be relaxed to as much as 30%. Because the non-orthogonality of grids has a much smaller impact on the waveform simulation accuracy, the model discretisation can be relatively flexible for fitting fluctuating boundaries within any complex problem. Consequently, seismic waveform inversion can explicitly include fluctuating interfaces within a subsurface velocity model.

## Introduction

Structural complexities such as the fluctuations of subsurface interfaces significantly influence seismic wavefields. For instance, fluctuating interfaces have both focusing and defocusing effects on seismic amplitudes. These interface-related amplitude effects can be exploited to reconstruct the interface geometry by inverting the seismic amplitudes^1–5^, and are also included implicitly in the waveform inversion of seismic reflection data^6^. Seismic reflection data, which are dominated by pre-critical reflected energy from subsurface contrasts in physical parameters, are suitable for structural imaging following seismic migration. However, these data can also be utilised in waveform inversions to quantitatively reconstruct velocity models for the subsurface of the Earth^6^. These reflection data are routinely recorded during explorations for hydrocarbons and mainly comprise of seismic P-wave data. Therefore, this paper uses an acoustic wave equation for a waveform simulation within an anisotropic medium.

To conduct an accurate waveform simulation for seismic waveform inversion, it is necessary to have an accurate representation of the subsurface interfaces within the numerical model, as the contrast in elastic properties across an interface has a dominant impact on the seismic wavefield. One of the suitable methods for waveform simulation is the finite-element method, since either an irregular surface or fluctuating interfaces can be described well by triangular gridding, including an adaptive meshing scheme^7,8^. The finite-element method is computationally expensive in comparison to a finite-difference method. A finite-difference method can be used with triangular grids^9^, but the errors in spatial derivative computations on unstructured grids were counteracted by using grids of higher node density. It is also computationally expensive.

A straightforward and cost-effective waveform simulation method is the finite-difference method with quadrilateral grids. In this paper, we adopt a body-fitted gridding scheme to partition each horizontal layer that is confined by two fluctuating interfaces in the model. Rao and Wang^10^ proposed strategies to produce grids for the irregular surface of Earth, to coincide with fluctuations of the surface while satisfying the pseudo-orthogonality condition. This pseudo-orthogonality condition is necessary to effectively avoid any scattering caused by artificial stair-type grids used within conventional rectangular gridding^11,12^. However, for a fluctuating interface, it is difficult to simultaneously adjust such grids on both sides of the interface, and to ensure that grids across the interface satisfy the pseudo-orthogonality condition. Nevertheless, we reveal in this paper that a summation-by-parts (SBP) discretisation approach may be effective for the finite-difference implementation of the wave equation. The SBP discretisation satisfies the SBP identity in Abel’s lemma^13^ that guarantees the stability of the finite-difference scheme^14–18^. We demonstrate that this SBP finite-difference method can cope with non-rectangular meshes in the vicinity of a fluctuating interface, and consequently achieve a desirable accuracy for the waveform simulation.

We focus on an acoustic waveform simulation and use a pseudo-acoustic wave equation for tilted transversely isotropic (TTI) media that are defined by two anisotropic parameters^19^. We extend this pseudo-acoustic wave equation to a model with surface and interface fluctuations. For a model that consists of an arbitrary number of interfaces, we apply the body-fitted gridding to each individual layer confined by two fluctuating interfaces. Non-rectangular meshes will become rectangular ones in the computational space through conformal mapping^20^. We reformulate the pseudo-acoustic wave equation and also derive the absorbing boundary condition, using the perfectly matched layer method^21^ in the computational space.

The quality of the meshing scheme in the physical space (before conformal mapping), determines the accuracy of the waveform simulation when utilising a finite-difference scheme. For instance, a low meshing quality reflects the characteristics of non-rectangular meshes, non-smoothness or abrupt mesh variations. The execution of the SBP finite-difference method leads to an improvement of the accuracy of the waveform simulation, by mitigating instability and coping with the low meshing qualities of body-fitted grids along the irregular surface of Earth, and along fluctuating interfaces.

## Wave equation in consideration of fluctuating interfaces

The pseudo-acoustic wave equation for TTI media is defined by two anisotropic parameters, namely, *ε* and *δ*, which measure the difference between the two axes of the elliptical wavefront and the deviation from a perfect elliptical shape respectively^22^. The pseudo-acoustic wave equation is^19^

1 $$\frac{{{\rm{\partial }}}^{2}}{{\rm{\partial }}{t}^{2}}(\begin{array}{c}p\\ q\end{array})=[\begin{array}{cc}{v}_{px}^{2}{H}_{x}+{v}_{sz}^{2}{H}_{z} & ({v}_{pz}^{2}-{v}_{sz}^{2}){H}_{z}\\ ({v}_{pn}^{2}-{v}_{sz}^{2}){H}_{x} & {v}_{sz}^{2}{H}_{x}+{v}_{pz}^{2}{H}_{z}\end{array}](\begin{array}{c}p\\ q\end{array}),$$

where *p* is the P wavefield, *q* is the auxiliary wavefield, *v*_*pz*_ and *v*_*sz*_ are the P-wave and SV-wave velocities respectively, along the axis of symmetry, *v*_*px*_ is the P-wave velocity perpendicular to the axis of symmetry, *v*_*pn*_ is the P-wave normal-moveout velocity, and *H*_*x*_ and *H*_*z*_ are two 2D differential operators, ${H}_{x}\equiv {\partial }^{2}/\partial {\hat{x}}^{2}$ and ${H}_{z}\equiv {\partial }^{2}/\partial {\hat{z}}^{2}$, with respect to the rotated coordinate system ($\hat{x}$, $\hat{z}$). The last two anisotropic velocities (*v*_*px*_, *v*_*pn*_) are related to the two anisotropy parameters *ε* and *δ* by^22^ ${v}_{px}={v}_{pz}\sqrt{1+2\varepsilon }$ and ${v}_{pn}={v}_{pz}\sqrt{1+2\delta }.$ Note that for the P-wave simulation, the SV-wave velocity *v*_*sz*_ remaining in eq. (1) does not have a significant effect on the wavefield.

**Figure 1:**
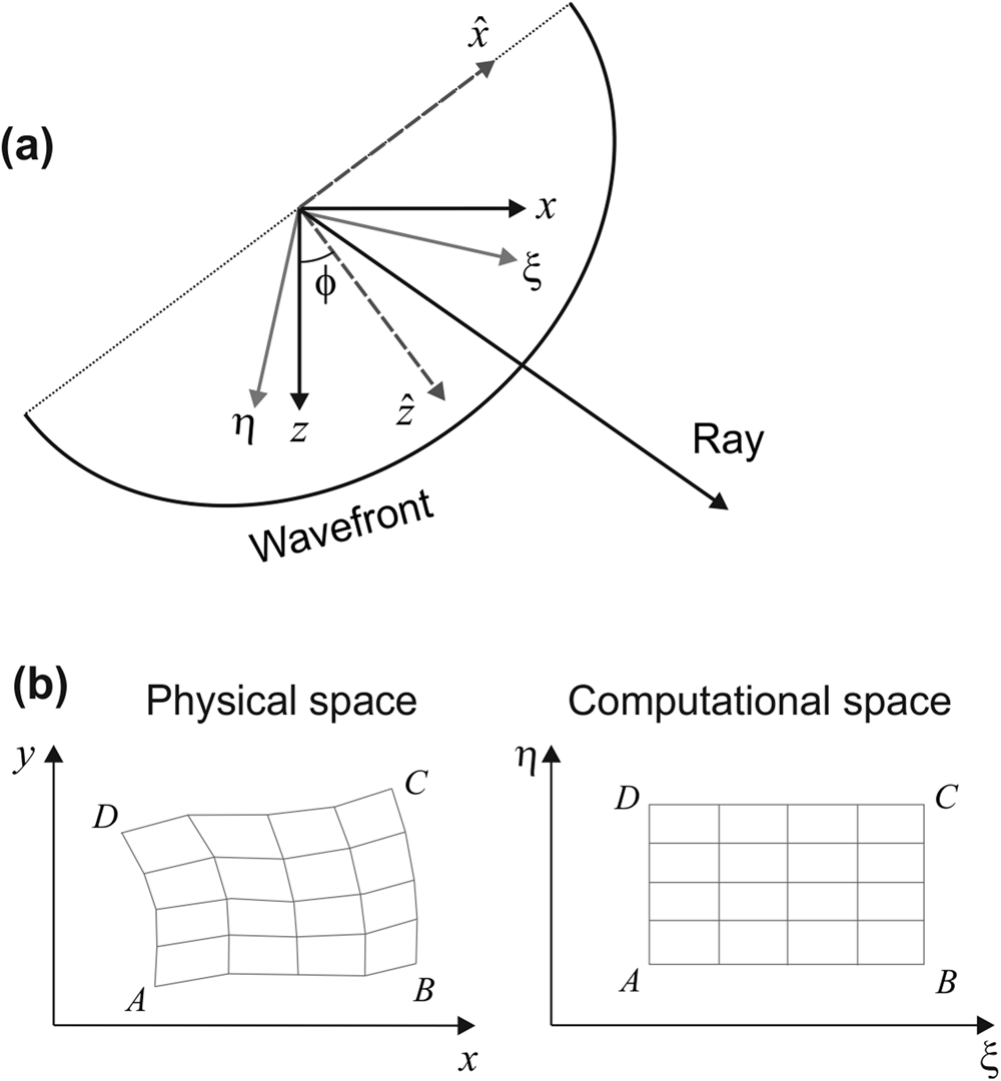
The TTI media and the computational space. **(a)** Within homogeneous TTI media, the dip angle of the axis of symmetry of a wavefront is *ϕ*. The ray vector points from the source to the receiver. **(b)** In consideration of fluctuations of the Earth’s surface and of subsurface interfaces, body-fitted grids are transformed from the Cartesian space (*x*, *y*) to a computational space (*ξ*, *η*).

In eq. (1), the two 2D differential operators in the Cartesian coordinate system (*x*, *z*) are^16^

2 $$\begin{array}{rcl}{H}_{x} & = & \frac{{\partial }^{2}}{\partial {x}^{2}}+\frac{{\partial }^{2}}{\partial {z}^{2}}-{H}_{z},\\ {H}_{z} & = & {\sin }^{2}\varphi \frac{{\partial }^{2}}{\partial {x}^{2}}+{\cos }^{2}\varphi \frac{{\partial }^{2}}{\partial {z}^{2}}+\,\sin \,2\varphi \frac{{\partial }^{2}}{\partial x\partial z},\end{array}$$

where *ϕ* is the dip angle of the axis of symmetry $\hat{z}$, measured anticlockwise from the vertical direction *z* (Fig. 1a). In consideration of fluctuations in the Earth’s surface and of the subsurface interfaces, we use a body-fitted gridding method^23^. For any horizontal layer confined by two fluctuating interfaces, we transform the differential operators in eq. (2), from the Cartesian coordinate system (*x*, *z*) to the computational space (*ξ*, *η*) (Fig. 1b).

In the computational space, the second-order spatial derivatives are

3 $$\begin{array}{rcl}\frac{{\partial }^{2}}{\partial {x}^{2}} & = & \frac{\partial }{\partial \xi }({\dot{\xi }}_{x}^{2}\frac{\partial }{\partial \xi })+\frac{\partial }{\partial \xi }({\dot{\xi }}_{x}{\dot{\eta }}_{x}\frac{\partial }{\partial \eta })+\frac{\partial }{\partial \eta }({\dot{\xi }}_{x}{\dot{\eta }}_{x}\frac{\partial }{\partial \xi })+\frac{\partial }{\partial \eta }({\dot{\eta }}_{x}^{2}\frac{\partial }{\partial \eta }),\\ \frac{{\partial }^{2}}{\partial {z}^{2}} & = & \frac{\partial }{\partial \xi }({\dot{\xi }}_{z}^{2}\frac{\partial }{\partial \xi })+\frac{\partial }{\partial \xi }({\dot{\xi }}_{z}{\dot{\eta }}_{z}\frac{\partial }{\partial \eta })+\frac{\partial }{\partial \eta }({\dot{\xi }}_{z}{\dot{\eta }}_{z}\frac{\partial }{\partial \xi })+\frac{\partial }{\partial \eta }({\dot{\eta }}_{z}^{2}\frac{\partial }{\partial \eta }),\\ \frac{{\partial }^{2}}{\partial x\partial z} & = & \frac{\partial }{\partial \xi }({\dot{\xi }}_{x}{\dot{\xi }}_{z}\frac{\partial }{\partial \xi })+\frac{\partial }{\partial \xi }({\dot{\xi }}_{x}{\dot{\eta }}_{z}\frac{\partial }{\partial \eta })+\frac{\partial }{\partial \eta }({\dot{\xi }}_{z}{\dot{\eta }}_{x}\frac{\partial }{\partial \xi })+\frac{\partial }{\partial \eta }({\dot{\eta }}_{x}{\dot{\eta }}_{z}\frac{\partial }{\partial \eta }),\end{array}$$

where ${\dot{\xi }}_{x}=\partial \xi /\partial x,$ ${\dot{\xi }}_{z}=\partial \xi /\partial z,\cdots ,$ are assumed to be known parameters. Subsequently, substituting these spatial derivatives into the 2D differential operators *H*_*x*_ and *H*_*z*_, we obtain the pseudo-acoustic wave eq. (1) in the computational space.

## The SBP finite-difference method

To avoid instabilities in numerical simulations caused both by low meshing precisions during body-fitted gridding and especially by strong variations in cell sizes, we propose an SBP finite-difference method that is unconditionally stable^24–26^. In this SBP method, the spatial derivatives in eq. (3) are represented as

4 $$\begin{array}{rcl}\frac{{\partial }^{2}}{\partial {x}^{2}} & = & {D}_{-}^{\xi }({E}_{1/2}^{\xi }({\dot{\xi }}_{x}^{2}){D}_{+}^{\xi })+{D}_{0}^{\xi }({\dot{\xi }}_{x}{\dot{\eta }}_{x}{D}_{0}^{\eta })+{D}_{0}^{\eta }({\dot{\xi }}_{x}{\dot{\eta }}_{x}{D}_{0}^{\xi })+{D}_{-}^{\eta }({E}_{1/2}^{\eta }({\dot{\eta }}_{x}^{2}){D}_{+}^{\eta }),\\ \frac{{\partial }^{2}}{\partial {z}^{2}} & = & {D}_{-}^{\xi }({E}_{1/2}^{\xi }({\dot{\xi }}_{z}^{2}){D}_{+}^{\xi })+{D}_{0}^{\xi }({\dot{\xi }}_{z}{\dot{\eta }}_{z}{D}_{0}^{\eta })+{D}_{0}^{\eta }({\dot{\xi }}_{z}{\dot{\eta }}_{z}{D}_{0}^{\xi })+{D}_{-}^{\eta }({E}_{1/2}^{\eta }({\dot{\eta }}_{z}^{2}){D}_{+}^{\eta }),\\ \frac{{\partial }^{2}}{\partial x\partial z} & = & {D}_{-}^{\xi }({E}_{1/2}^{\xi }({\dot{\xi }}_{x}{\dot{\xi }}_{z}){D}_{+}^{\xi })+{D}_{0}^{\xi }({\dot{\xi }}_{x}{\dot{\eta }}_{z}{D}_{0}^{\eta })+{D}_{0}^{\eta }({\dot{\xi }}_{z}{\dot{\eta }}_{x}{D}_{0}^{\xi })+{D}_{-}^{\eta }({E}_{1/2}^{\eta }({\dot{\eta }}_{x}{\dot{\eta }}_{z}){D}_{+}^{\eta }),\end{array}$$

where ${D}_{+}^{r}(p)={h}^{-1}({p}_{j+1}-{p}_{j})$ and ${D}_{-}^{r}(p)={h}^{-1}({p}_{j}-{p}_{j-1})$ are the forward and backward differential operators, respectively, ${D}_{0}^{r}(p)={h}^{-1}({p}_{j+1}-{p}_{j-1})/2$ is the central differential operator, ${E}_{1/2}^{\xi }({p}_{j})=({p}_{j}+{p}_{j+1})/2$ is an average value for *p*_*j* + 1/2_, and *h* is the spatial sampling interval of either *ξ* or *η*.

The differential operators *H*_*x*_ and *H*_*z*_ in eq. (2) are then expressed explicitly as

5 $$\begin{array}{rcl}{H}_{x} & = & {D}_{-}^{\xi }({E}_{1/2}^{\xi }({\dot{\xi }}_{x}^{2}){D}_{+}^{\xi })+{D}_{0}^{\xi }({\dot{\xi }}_{x}{\dot{\eta }}_{x}{D}_{0}^{\eta })+{D}_{0}^{\eta }({\dot{\xi }}_{x}{\dot{\eta }}_{x}{D}_{0}^{\xi })+{D}_{-}^{\eta }({E}_{1/2}^{\eta }({\dot{\eta }}_{x}^{2}){D}_{+}^{\eta })\\  &  & +{D}_{-}^{\xi }({E}_{1/2}^{\xi }({\dot{\xi }}_{z}^{2}){D}_{+}^{\xi })+{D}_{0}^{\xi }({\dot{\xi }}_{z}{\dot{\eta }}_{z}{D}_{0}^{\eta })+{D}_{0}^{\eta }({\dot{\xi }}_{z}{\dot{\eta }}_{z}{D}_{0}^{\xi })+{D}_{-}^{\eta }({E}_{1/2}^{\eta }({\dot{\eta }}_{z}^{2}){D}_{+}^{\eta })\\  &  & -{H}_{z},\\ {H}_{z} & = & {\sin }^{2}\varphi [{D}_{-}^{\xi }({E}_{1/2}^{\xi }({\dot{\xi }}_{x}^{2}){D}_{+}^{\xi })+{D}_{0}^{\xi }({\dot{\xi }}_{x}{\dot{\eta }}_{x}{D}_{0}^{\eta })+{D}_{0}^{\eta }({\dot{\xi }}_{x}{\dot{\eta }}_{x}{D}_{0}^{\xi })+{D}_{-}^{\eta }({E}_{1/2}^{\eta }({\dot{\eta }}_{x}^{2}){D}_{+}^{\eta })]\\  &  & +{\cos }^{2}\varphi [{D}_{-}^{\xi }({E}_{1/2}^{\xi }({\dot{\xi }}_{z}^{2}){D}_{+}^{\xi })+{D}_{0}^{\xi }({\dot{\xi }}_{z}{\dot{\eta }}_{z}{D}_{0}^{\eta })+{D}_{0}^{\eta }({\dot{\xi }}_{z}{\dot{\eta }}_{z}{D}_{0}^{\xi })+{D}_{-}^{\eta }({E}_{1/2}^{\eta }({\dot{\eta }}_{z}^{2}){D}_{+}^{\eta })]\\  &  & +\,\sin \,2\varphi [{D}_{-}^{\xi }({E}_{1/2}^{\xi }({\dot{\xi }}_{x}{\dot{\xi }}_{z}){D}_{+}^{\xi })+{D}_{0}^{\xi }({\dot{\xi }}_{x}{\dot{\eta }}_{z}{D}_{0}^{\eta })+{D}_{0}^{\eta }({\dot{\xi }}_{z}{\dot{\eta }}_{x}{D}_{0}^{\xi })+{D}_{-}^{\eta }({E}_{1/2}^{\eta }({\dot{\eta }}_{x}{\dot{\eta }}_{z}){D}_{+}^{\eta })].\end{array}$$

We have thus completed the discretisation of the pseudo-acoustic wave equation using the SBP finite-difference method. Due to the presence of fluctuating interfaces within the model, we performed this discretisation in the computational space (*ξ*, *η*).

## Results

After the model has been partitioned using body-fitted grids, the meshing quality will ultimately determine the numerical accuracy of the waveform simulation with a finite-difference method. Conventional finite-difference methods for isotropic media impose strict requirements on the qualities of body-fitted grids^10^. The requirements for such grids include two aspects: the acute angle of the grids should be >67°, and the rate of change in the cell size between neighbouring grids should be <5%.

**Figure 2:**
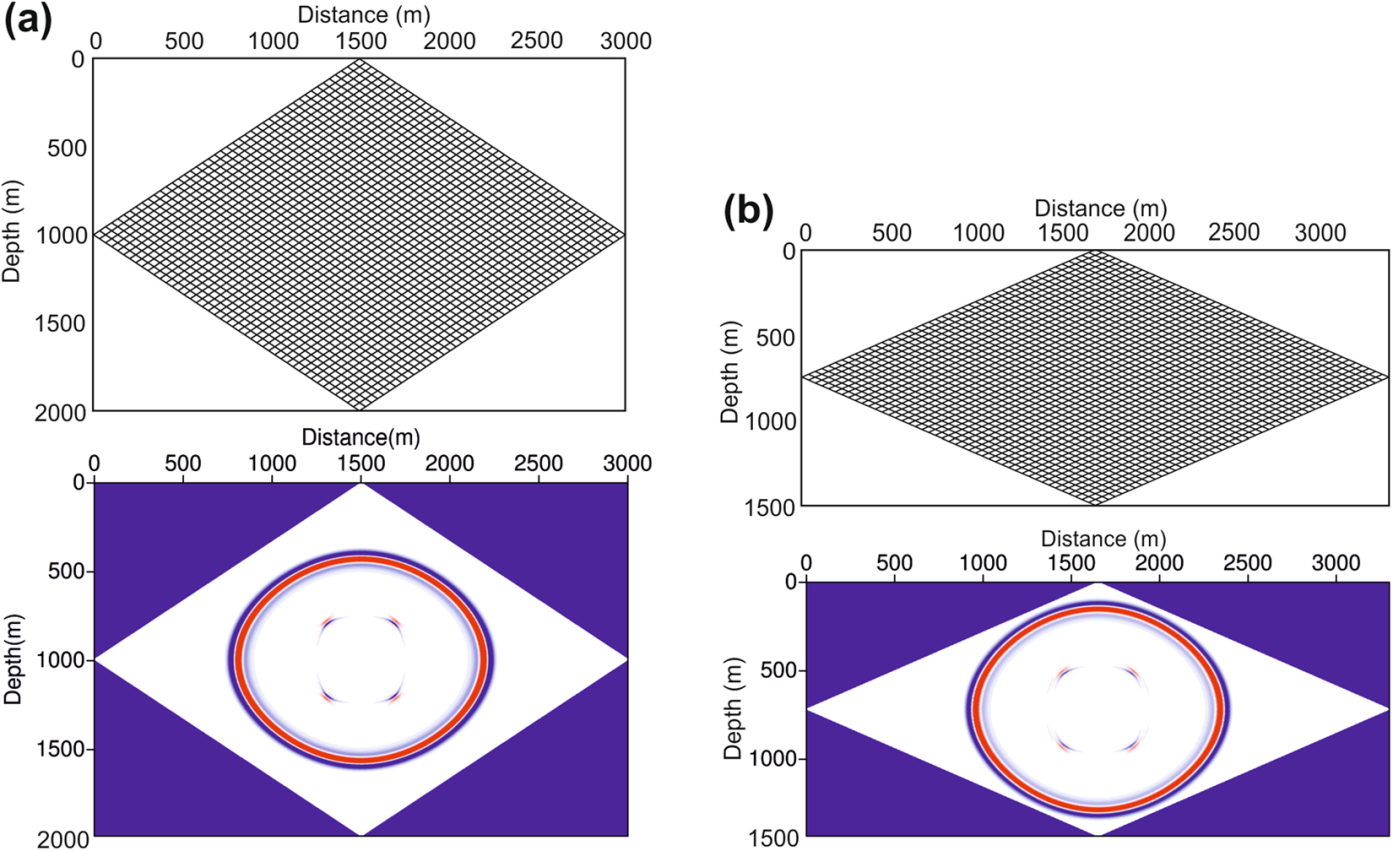
Seismic wave simulation in parallelogram grids. **(a)** Parallelogram grids with an acute angle of 67° and a snapshot of the wave propagation at 195 ms. **(b)** Parallelogram grids with an acute of angle 47° and a snapshot of the wave propagation at 195 ms.

However, we discover that the grid orthogonality requirement can be relaxed further to an acute angle of >47° (from an ideal case of 90°) when using the SBP finite-difference method for a waveform simulation, following which the simulation can maintain stability and a reliable performance accuracy. Figure 2a and b show two meshes with acute angles of 67° and 47° respectively. Snapshots of the wavefront propagation acquired at 195 ms clearly indicate that both cases (i.e., with mesh acute angles of either 67° or 47°) show reasonable performance accuracies.

**Figure 3:**
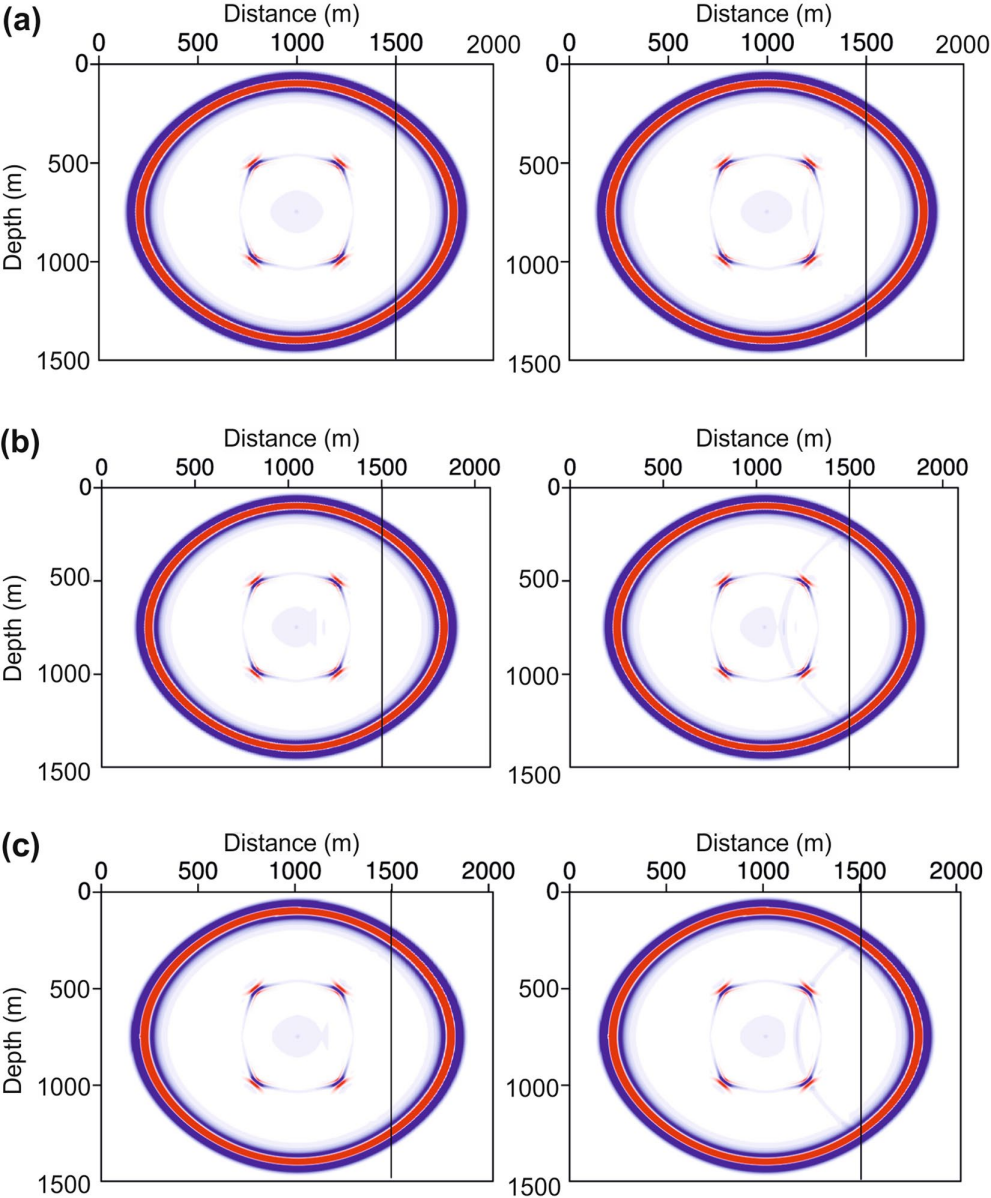
Wavefield simulation in grids with different change rates of the cell size. **(a)** The rate of change in the cell size is 5% at the marked (solid line) distance of 1500 m. **(b)** The rate of change is 30% at the marked distance. **(c)** The rate of change is 40% at the marked distance. The left-hand column illustrates snapshots (at 240 ms) of the SBP finite-difference method, and the right-hand column illustrates snapshots of the conventional finite-difference method.

The tolerance to the rate of change in the cell size is also improved from 5% to 30% during the waveform simulation when using the SBP finite-difference method. Figure 3 compares snapshots acquired at 240 ms, using both the SBP finite-difference method (left-hand column) and the conventional finite-difference method (right-hand column). At a marked distance (solid line at 1500 m), the horizontal cell size on the left-hand side is a constant of 3 m, whereas the horizontal cell sizes on the right-hand side are 3.15 m, 3.9 m, and 4.2 m, corresponding to 5%, 30%, and 40% rates of change in the cell size respectively, for the three panels from the top to the bottom (Fig. 3). When using the conventional finite-difference method, even a 5% cell-size change (snapshot on the right-hand column of Fig. 3a) can produce an artificial reflection boundary within the homogeneous media. However, when using the SBP finite-difference method, only a change rate as large as 40% (snapshot on the left-hand column of Fig. 3c) generates an artificial reflection boundary.

**Figure 4:**
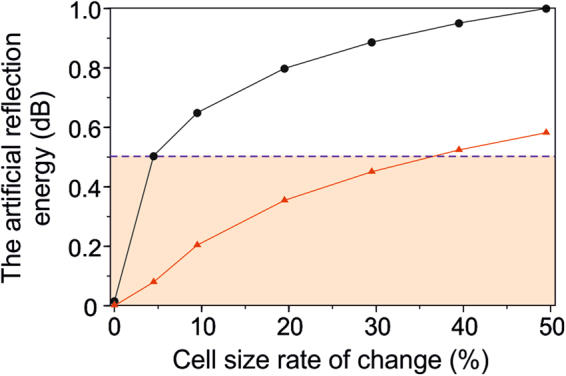
The artificial reflection energy (in dB). The artificial reflections are caused by different rates of change in the cell size. The solid dots show the artificial reflection energies from the conventional finite-difference method, and the triangles represent the artificial reflection energies from the SBP finite-difference method. The shaded zone represents the sufficiently small artificial reflection energies that are less than 0.5 dB (dashed line) for different rates of change in the cell size.

Figure 4 graphically summarises the artificial reflection energies that are caused by the different rates of change in the cell size. The two curves correspond to the conventional finite-difference method (solid dots) and the SBP finite-difference method (triangles). The SBP method generally exhibits much smaller reflection energies, as a consequence of the cell-size changes. Nevertheless, we establish a threshold at 0.5 dB (dashed line in Fig. 4), so that both the 5% and 30% cell-size rates of change for the respective schemes have sufficiently small amounts of artificial reflection energies (shaded zone).

**Figure 5:**
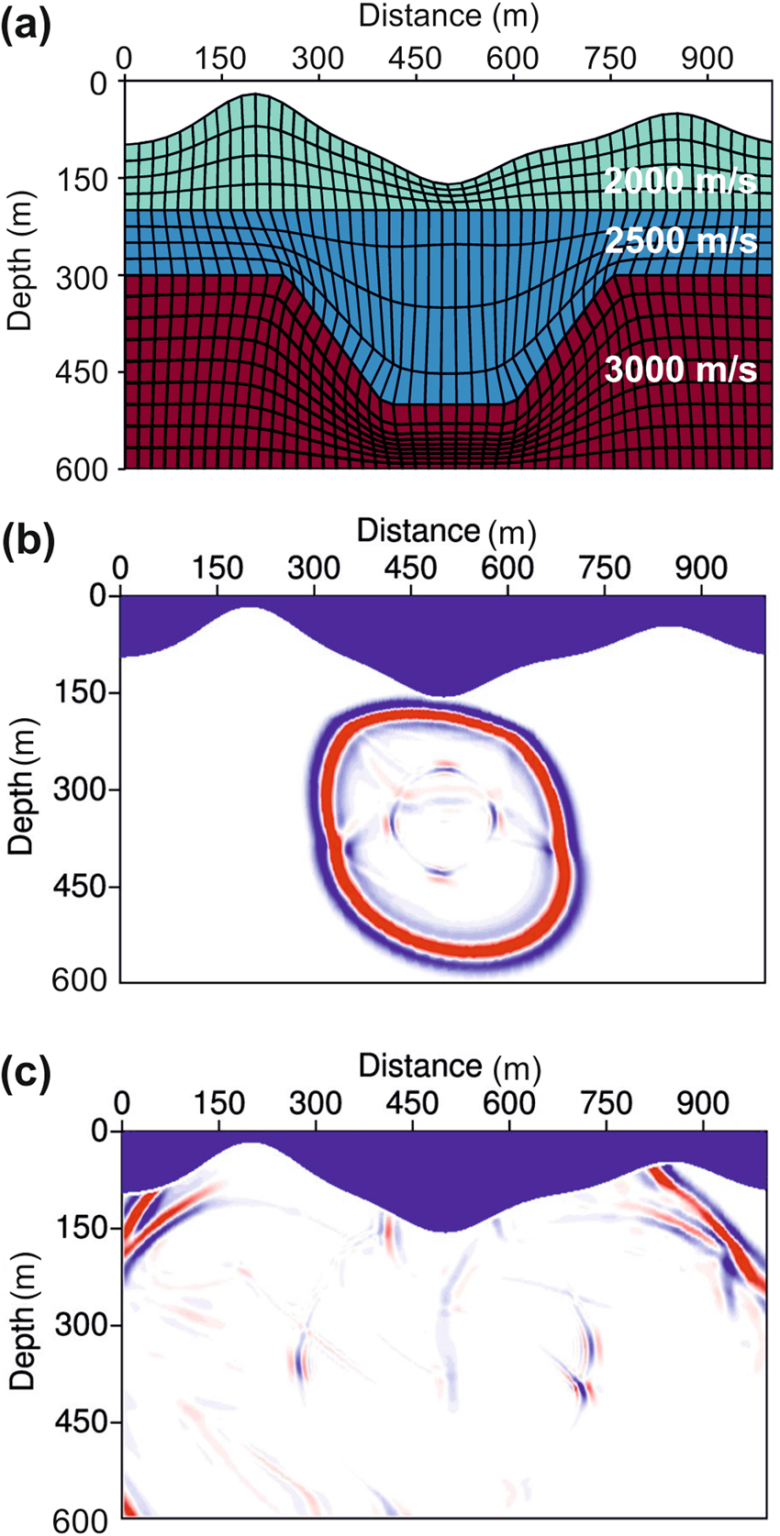
Wave simulation in a model with a fluctuating surface and interfaces. **(a)** A three-layer velocity model that is masked by body-fitted gridding. The body-fitted grids coincide well with both the irregular surface and the fluctuating interfaces of Earth. **(b)** Snapshot of the wavefield at 85 ms. **(c)** Snapshot of the wavefield at 207 ms.

Figure 5 shows an example of a waveform simulation in a three-layer model with the irregular surface of the Earth and fluctuating subsurface interfaces. The P-wave velocities of the three layers from the top to the bottom are *v*_*pz*_ = 2000, 2500, and 3000 m/s. The S-wave velocity ${v}_{sz}={v}_{pz}/\sqrt{3}$ is a constant, and the two anisotropic parameters *ε* = 0.24 and *δ* = 0.1 are also a constant. The body-fitted grids (Fig. 5a) are generated layer-by-layer in order to effectively avoid artificial scattering during the conventional rectangular gridding process, and the grids coincide well with both the irregular surface of the Earth and the fluctuating interfaces.

A classic method is to smooth out the existence of a gently dipping interface by averaging the medium parameters within a grid, which represents equivalently to a tilted two-layer media separated by a locally straight-lined interface^27^. This method keeps working on rectangular grids, and does not need the conformal mapping which we need here when using body-fitted gridding. However, this method of employing an averaging scheme does not work for the acoustic wave simulation with a free surface, in which the top side of the fluctuating surface is vanished. It also breaks down for the elastic wave simulation with a fluid/solid interface, because of the vanished shear modulus in fluid.

The snapshots (Fig. 5b and c) indicate that the SBP finite-difference method, which has been extended to simulate the wave equation for TTI media, is stable for strong velocity variations and complicated model structures. While this method evidently works for the case with a fluctuating free surface, it will work also for the elastic wave simulation with a fluid/solid interface, as it avoids averaging an elastic medium with a vanished shear modulus in fluid.

## Conclusions

Body-fitted gridding schemes are effective for partitioning numerical models within seismic waveform simulations when considering complex fluctuations of both the Earth’s surface and the subsurface interfaces. However, when these schemes are used to fit these complex fluctuations, non-rectangular meshes are inevitably generated across the interfaces, and the resulting grids cannot always satisfy the pseudo-orthogonality condition. The non-orthogonality of the meshing will degrade the precision of the mesh, and reduce the accuracy of the waveform simulation. This paper revealed that when using the proposed SBP finite-difference method, non-rectangular meshes can be included within the implementation of a waveform simulation. The acute angle of the grids may be relaxed to as low as 47° (from an ideal case of 90°), and the rate of change in the cell size could be relaxed to as much as 30%. By contrast, these two quantities are 67° and 5% respectively, when using the conventional finite-difference method^5^. Therefore, when using the SBP finite-difference method, the body-fitted grids can be more flexible to fit fluctuating boundaries that confine any subsurface layer, and the non-orthogonality of the meshing scheme has a smaller impact on the accuracy of the waveform simulation. Therefore, seismic waveform inversion for velocity imaging endeavours is capable of explicitly including fluctuating interfaces within a subsurface model.
